# Clash of the calculators: External validation of prostate cancer risk calculators in men undergoing mpMRI and transperineal biopsy

**DOI:** 10.1002/bco2.58

**Published:** 2021-03-03

**Authors:** G. Wei, B. D. Kelly, B. Timm, M. Perera, D. J. Lundon, G. Jack, D. M. Bolton

**Affiliations:** ^1^ Department of Surgery Austin Health The University of Melbourne Melbourne VIC Australia; ^2^ North Eastern Urology Melbourne VIC Australia; ^3^ Olivia Newton‐John Cancer and Wellness Centre Austin Health Heidelberg VIC Australia; ^4^ Faculty of Medicine University of Queensland Brisbane QLD Australia; ^5^ Department of Urology Icahn School of Medicine Mount Sinai Hospitals New York NY USA

**Keywords:** biopsy, ERSPC, mag nomogram, netic resonance imaging, PBCG, prostate cancer

## Abstract

**Objective:**

To compare the accuracy of the European Randomized Study of Screening for Prostate Cancer (ERSPC) RC, MRI‐ERSPC‐RC, and Prostate Biopsy Collaborative Group (PBCG) RC in patients undergoing transperineal prostate biopsy.

**Patients and methods:**

We identified 392 patients who underwent mpMRI before transperineal prostate biopsy across multiple public and private institutions between January 2017 and August 2019. The estimated probabilities of detecting PCa and significant PCa were calculated using the MRI‐ERSPC‐RC, ERSPC‐RC, and PBCG‐RC. Receiver operating characteristic (ROC) curves for each calculator were generated and the area underneath the curve (AUC) was compared. Calibration and clinical utility were assessed with calibration plots and decision curve analysis, respectively.

**Results:**

PCa was detected in 285 patients (72.7%) with significant PCa found in 200 patients (51.1%). ROC curve analysis found the MRI‐ERSPC‐RC outperformed the ERSPC‐RC and PBCG‐RC. For the prediction of PCa, the AUC was 0.756, 0.696, and 0.675 for the MRI‐ERSPC‐RC, ERSPC‐RC, and PBCG‐RC, respectively. The AUC for the prediction of significant PCa was 0.803, 0.745, and 0.746 for the MRI‐ERSPC‐RC, ERSPC‐RC, and PBCG‐RC, respectively.

**Conclusions:**

Our study validated the ERSPC‐RC, MRI‐ERSPC‐RC, and PBCG‐RC in a cohort undergoing transperineal prostate biopsy with the MRI‐ERSPC‐RC performing the best. These RCs may enable improved shared decision making and help to guide patient selection for biopsy.

## 1 INTRODUCTION

Prostate cancer (PCa) is the second most commonly diagnosed cancer in men worldwide with almost 1.3 million new cases in 2018.^1^ Traditional approaches to patient selection for prostate biopsy utilizing prostate‐specific antigen (PSA) and digital rectal examination (DRE) have contributed to many men having negative biopsies as well as an increased detection of low‐risk PCa.^2^ Subsequently, many men undergo prolonged follow‐up with significant costs and burdens to the patient as well as the healthcare system.

In recent years, multi‐parametric Magnetic Resonance Imaging (mpMRI) has become increasingly accessible. There is expanding evidence that pre‐diagnostic mpMRI helps to optimize patient selection for biopsy by reducing unnecessary prostate biopsies and improving diagnostic accuracy.^3^, ^4^, ^5^ Consequently, standard clinical practice has changed worldwide with many healthcare systems now offering men an mpMRI in the initial workup of PCa prior to their biopsy.

In an attempt to mitigate unnecessary biopsies, several pre‐diagnostic risk calculators have been developed to optimize patient selection prior to biopsy. The European Randomized Study of Screening for Prostate Cancer (ERSPC) and Prostate Biopsy Collaborative Group (PBCG) risk calculators (RC) aim to estimate the likelihood of any PCa and clinically significant PCa.^6^, ^7^ With its rise in popularity, mpMRI has been incorporated into the latest ERSPC‐RC together with PSA, history of negative prostate biopsy, DRE, prostate volume, and age.^6^ The aim of our study was to assess the validity of three RCs; the MRI‐ERSPC‐RC, the previous ERSPC‐RC, and the PBCG‐RC. We aimed to compare their respective performances in order to identify which RC provides the superior model in optimizing patient selection for biopsy.

## 2 PATIENTS AND METHODS

Patients with a clinical suspicion of PCa who underwent mpMRI followed by a transperineal prostate biopsy between January 2017 and August 2019 were identified from a prospectively maintained database. This database comprised patients from multiple institutions encompassing both the public and private healthcare system. Patients with previously diagnosed PCa or without a pre‐biopsy mpMRI were excluded. Furthermore, patients whose age, PSA level, or prostate volume precluded the use of the ERSPC‐RC or PBCG‐RC were excluded. Approval for this project was granted by our institution's Human Research Ethics Committee.

Transperineal prostate biopsy was performed by a urologist under general anesthesia or sedation with the patient in lithotomy position using a bi‐planar ultrasound transducer probe (BK Medical, Peabody, USA) in the rectum and an 18g x 22cm biopsy needle (Bard Max Core Needle, Bard, USA). Prostate mapping was performed in 5‐10 mm increments utilizing a brachytherapy template grid (Accucare Template grid, Civco Medical Solutions, UK). All patients underwent systematic biopsy in addition to targeted biopsies from areas of concern identified by mpMRI. The template used and total number of cores taken were in accordance with the Ginsburg protocol.^8^ Specimens were assessed by genitourinary pathologists using the International Society of Urological Pathology (ISUP) Grade Group system.^9^ In the current study, clinically significant cancer was defined as ISUP Grade Group ≥ 2.

All mpMRIs were performed using a 3 Tesla MRI scanner including diffusion weighted, dynamic contrast enhanced T1 and T2 weighted imaging and reported as per the Prostate Imaging—Reporting and Data System (PI‐RADS) by a group of radiologists specializing in uro‐oncology.^10^ All mpMRIs and prostate biopsies were reviewed at a urology multidisciplinary team meeting and reviewed by dedicated uro‐pathologists and uro‐radiologists.

Medical records were reviewed and data obtained as guided by the PBCG and ERSPC calculators, including race, age, pre‐biopsy PSA, family history, history of previous biopsy, DRE findings, prostate volume, PI‐RADS score, and histopathology results.

The risk of any PCa diagnosis and risk of high‐grade disease were calculated and defined utilizing the PBCG‐RC, MRI‐ERSPC‐RC, and ERSPC‐RC online calculators.^11^, ^12^ The ERSPC‐RCs calculated the risk for overall cancer detection and the detection of significant cancer (ISUP Grade Group ≥ 2). The PBCG‐RC calculated the risk for low‐ and high‐risk cancer (ISUP Grade Group ≥ 2) which were added together to calculate the result for overall cancer detection. R statistical software, version 3.6.3 (R Core Team, Vienna, Austria) and Minitab, version 19.2 (LLC, Pennsylvania, USA) were used to compare the respective risk calculations with the final biopsy histological grade.

To quantify the discriminative ability of the MRI‐ERSPC‐RC, ERSPC‐RC, and PBCG‐RC, receiver operating characteristic (ROC) curves for each RC were generated and plotted as the false negative rate (1‐specificity) vs sensitivity.^13^ An AUC of 0.5 was considered to demonstrate no discrimination, 0.5‐0.7 was considered poor discrimination, 0.7‐0.8 was considered acceptable discrimination, 0.8‐0.9 was considered excellent discrimination, and greater than 0.9 was considered outstanding.^14^ Areas underneath the ROC curve (AUC) were calculated for the respective calculators and compared DeLong's test.^15^ Calibration plots were computed by comparing observed proportions of cancer to mean calculated risks by the respective risk calculator deciles observed in the cohort for overall cancer detection and significant cancer risk.^16^ The Hosmer‐Lemeshow Chi square test was used to compare the observed rates to predicted risks across the deciles for each calculator. For this test, a *P* < .05 indicates a poor agreement between predicted risks and actual observed risk. Decision curve analysis was performed to assess for the gain derived from the respective risk calculator over the corresponding net benefit curves of referring no patients or all patients to biopsy.^17^

## 3 RESULTS

Eight hundred and sixty‐four patients who underwent transperineal prostate biopsy were identified. One hundred and nineteen patients had previously diagnosed PCa and 283 patients did not undergo pre‐biopsy mpMRI. A further 70 patients were unable to have their risk estimated using the ERSPC‐RC or the PBCG‐RC. A total of 392 patients met our final inclusion criteria. The median age, PSA, and prostate volume were 64 years, 6 ng/ml, and 43 ml, respectively. Eighty‐one patients (20.7%) had a positive family history for PCa, 42 patients (10.7%) had a previous negative biopsy, and 103 patients (26.3%) had an abnormal or suspicious DRE.

The majority of patients had an equivocal or suspicious mpMRI with 308 patients (78.6%) having an mpMRI of PI‐RADS ≥ 3. Overall, 285 men were diagnosed with PCa of any grade (72.7%) with 200 men having ISUP Grade Group ≥ 2 disease (51.1%). Patient demographics and tumor characteristics are summarized in greater detail in Table 1.

**TABLE 1:** Patient and tumor characteristics

Characteristic	n = 392
Age (years)	64 (50‐69)
PSA (ng/ml)	6 (2‐9)
Prostate volume (ml)	43 (15‐62)
Positive family history	81 (20.7%)
Previous negative biopsy	42 (10.7%)
DRE	
Benign	289 (73.7%)
Suspicious	103 (26.3%)
PI‐RADS	
1‐2	84 (21.4%)
3	67 (17.1%)
4	171 (43.6%)
5	70 (17.9%)
Histopathology	
No PCa	107 (27.3%)
Grade Group 1	85 (21.7%)
Grade Group 2	103 (26.3%)
Grade Group 3	54 (13.8%)
Grade Group 4	20 (5.1%)
Grade Group 5	23 (5.9%)

Note

Data are presented as median (IQR) or n (%).

Overall, PI‐RADS score predicted PCa of any grade and clinically significant PCa. A total of 56.7% of PI‐RADS 3 lesions, 82.5% of PI‐RADS 4 lesions, and 91.4% of PI‐RADS 5 lesions had a biopsy confirming PCa. 26.9% of patients with PI‐RADS 3 mpMRIs had a biopsy revealing Grade Group ≥ 2 disease. In contrast, 61.4% of PI‐RADS 4 lesions and 82.9% of PI‐RADS 5 lesions had Grade Group ≥ 2 disease. These results are described in more detail in Table 2.

**TABLE 2:** Histopathological results of patients who underwent diagnostic transperineal prostate biopsy stratified by PI‐RADS score

	PI‐RADS ≤ 2	PI‐RADS 3	PI‐RADS 4	PI‐RADS 5	Total
	n = 84	n = 67	n = 171	n = 70	n = 392
Benign	42 (50%)	29 (43.3%)	30 (17.5%)	6 (8.6%)	107 (27.3%)
Total cancer	42 (50%)	38 (56.7%)	141 (82.5%)	64 (91.4%)	285 (72.7%)
Grade Group 1	23 (27.4%)	20 (29.8%)	36 (21.0%)	6 (8.6%)	85 (21.7%)
Grade Group 2	14 (16.6%)	13 (19.4%)	55 (32.2%)	21 (30%)	103 (26.3%)
Grade Group 3	5 (6.0%)	4 (6.0%)	28 (16.4%)	17 (24.3%)	54 (13.8%)
Grade Group 4	0 (0%)	0 (0%)	15 (8.8%)	5 (7.1%)	20 (5.1%)
Grade Group 5	0 (0%)	1 (1.5%)	7 (4.1%)	15 (21.4%)	23 (5.8%)

Note

Percentages expressed as a proportion of the total in each respective PI‐RADS score.

**FIGURE 1:**
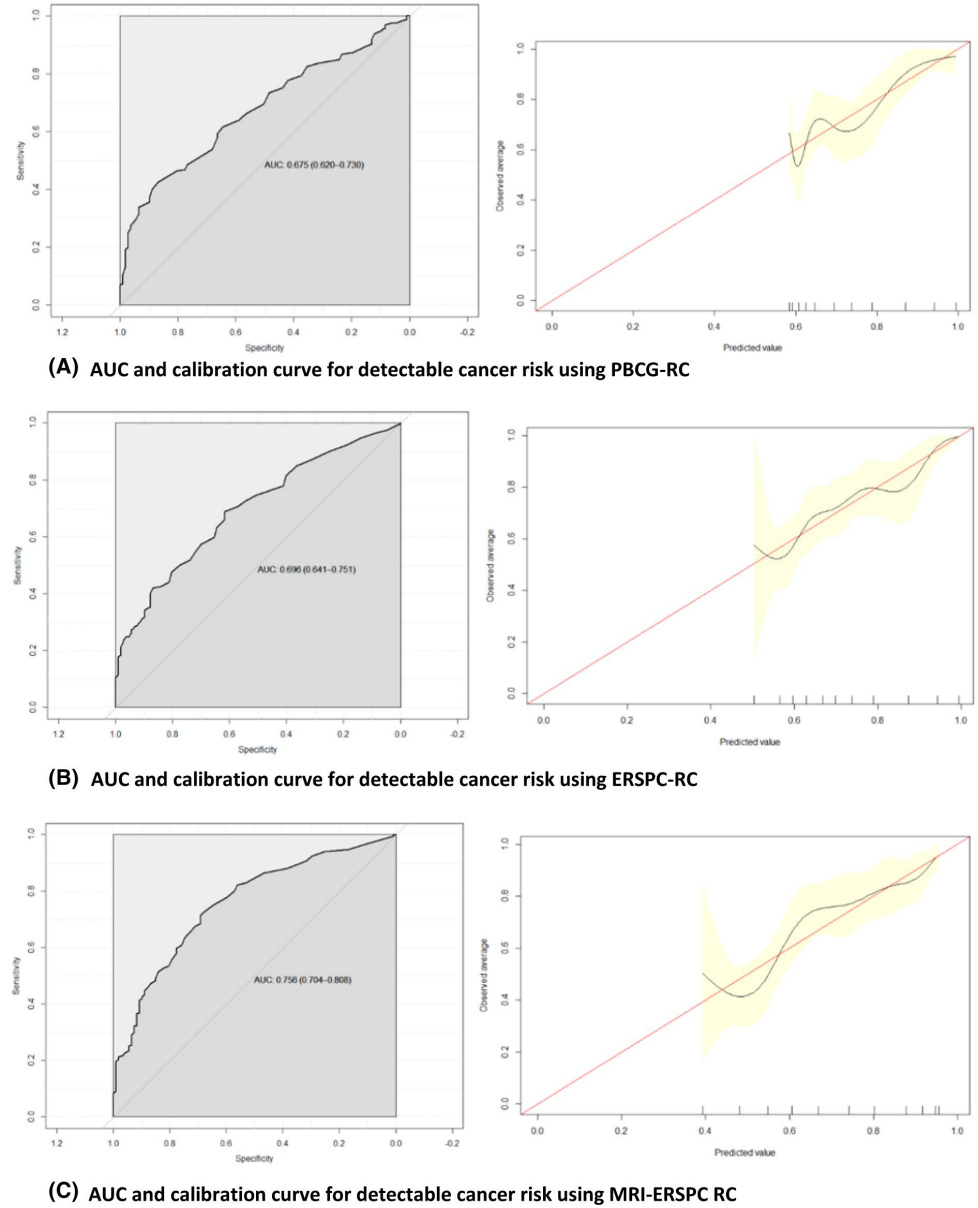
AUC and calibration curves for detectable cancer risk. (A) AUC and calibration curve for detectable cancer risk using PBCG‐RC. (B) AUC and calibration curve for detectable cancer risk using ERSPC‐RC. (C) AUC and calibration curve for detectable cancer risk using MRI‐ERSPC RC

**FIGURE 2:**
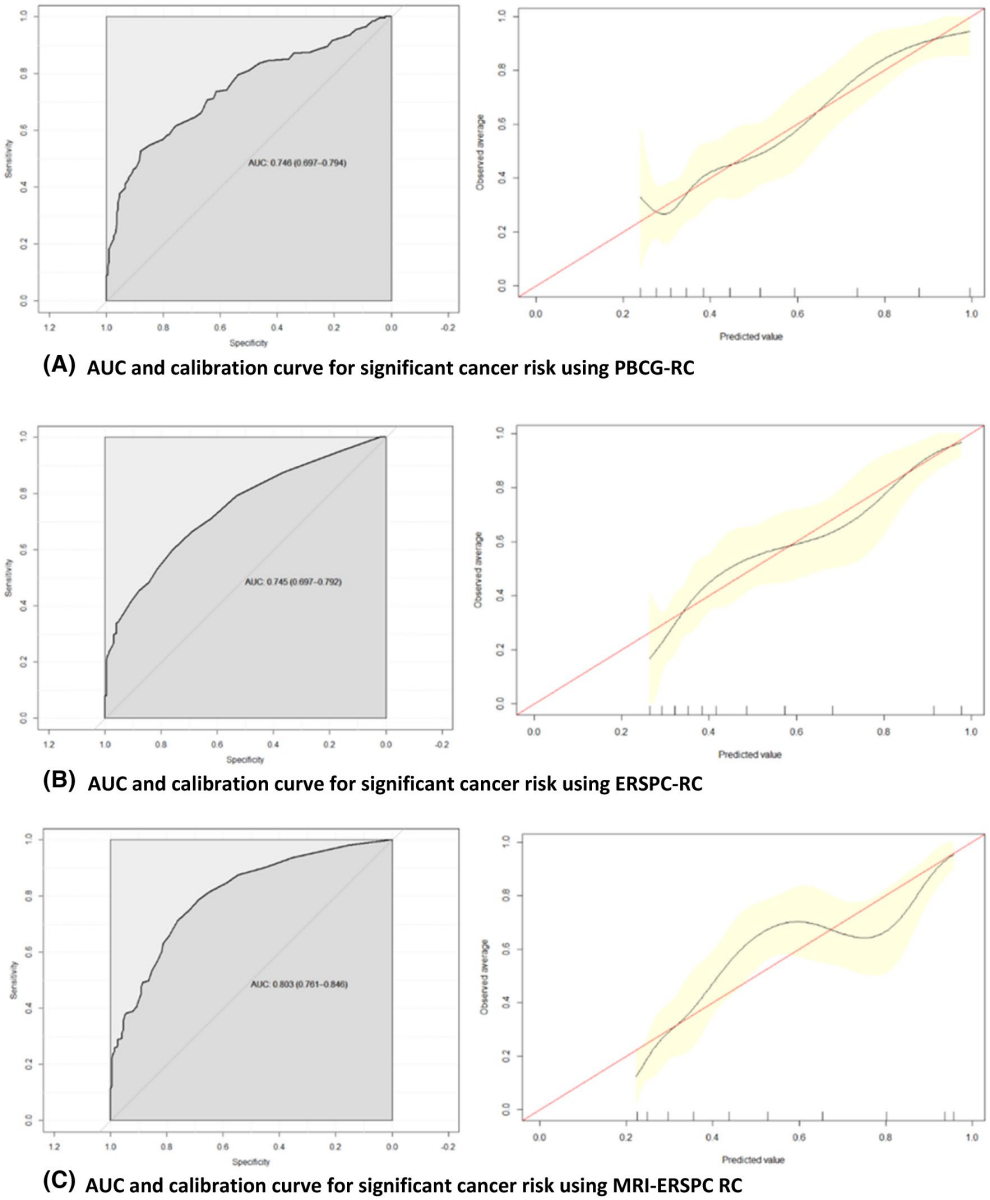
AUC and calibration curves for significant cancer risk. (A) AUC and calibration curve for significant cancer risk using PBCG‐RC. (B) AUC and calibration curve for significant cancer risk using ERSPC‐RC. (C) AUC and calibration curve for significant cancer risk using MRI‐ERSPC RC

The AUC value for prediction of cancer of any grade was 0.696 for ERSPC‐RC, 0.756 for MRI‐ERSPC‐RC, and 0.675 for PBCG‐RC. The discriminative ability of the MRI‐ERSPC‐RC was statistically superior to the ERSPC‐RC (*P* = 0.011) and PBCG‐RC (*P* = 0.003). There was no statistical difference between the ERSPC‐RC and PBCG‐RC (*P* = 0.469). For predicting Grade Group ≥ 2 disease, the AUC was 0.745 for ERSPC‐RC, 0.803 for MRI‐ERSPC‐RC, and 0.746 for PBCG‐RC. The discriminative ability of the MRI‐ERSPC‐RC was superior to the ERSPC‐RC (*P* = 0.010) and PBCG‐RC (*P* = 0.012). The ERSPC‐RC and PBCG‐RC were similar in their ability to predict Grade Group ≥ 2 disease (*P* = 0.964). These results are depicted in Tables 3 and 4 and Figures 1 and 2.

**TABLE 3:** Comparison of the predictive accuracy of the ERSPC‐RC, MRI‐ERSPC‐RC, and PBCG‐RC

	ERSPC‐RC AUC	MRI‐ERSPC‐RC AUC	PBCG‐RC AUC
Overall cancer detection	0.696	0.756	0.675
Significant cancer detection	0.745	0.803	0.746

**TABLE 4:** Comparison between the AUC of the ERSPC‐RC, MRI‐ERSPC‐RC, and PBCG‐RC using DeLong's test

	ROC Curve 1	ROC Curve 2	*P*‐value
Overall cancer detection	MRI‐ERSPC‐RC	ERSPC‐RC	.011
	MRI‐ERSPC‐RC	PBCG‐RC	.003
	ERSPC‐RC	PBCG‐RC	.469
Significant cancer detection (Grade Group ≥ 2)	MRI‐ERSPC‐RC	ERSPC‐RC	.010
	MRI‐ERSPC‐RC	PBCG‐RC	.012
	ERSPC‐RC	PBCG‐RC	.964

**FIGURE 3:**
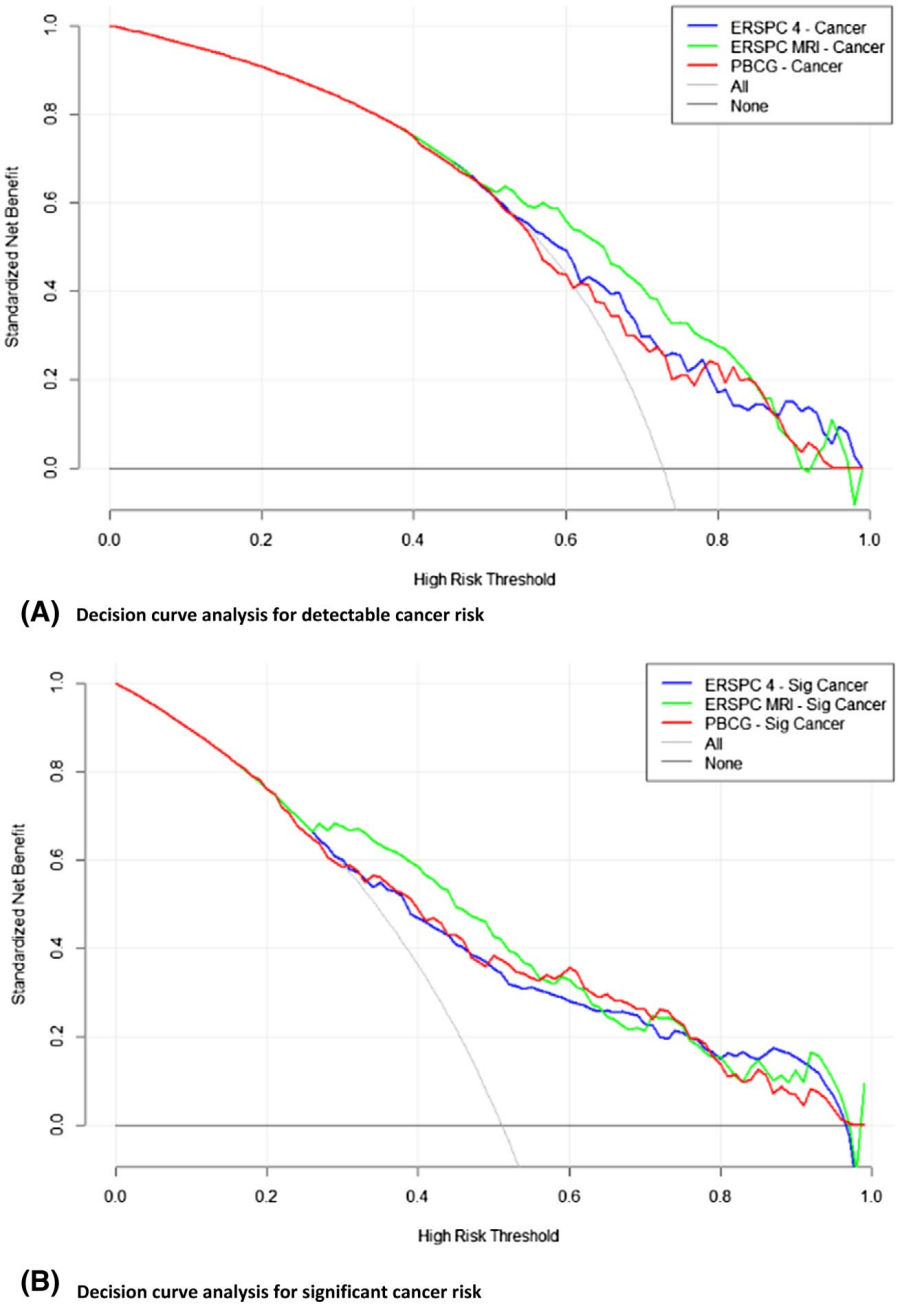
Decision curve analysis for detectable cancer risk and significant cancer risk. (A) Decision curve analysis for detectable cancer risk. (B) Decision curve analysis for significant cancer risk

Figure 3 demonstrates the results of decision curve analysis with separate decision curves for each RC for detectable cancer risk (Figure 3a) and significant cancer risk (Figure 3b). For both figures, the line at *y* = 0 represents the decision curve if no biopsies were performed and the gray line represents the decision curve for performing a biopsy on all patients. The green, blue, and red lines represent the benefit of using each respective RCs to determine which patient to biopsy. The MRI‐ERSPC‐RC was superior to the ERSPC‐RC and the PBCG‐RC for the prediction of both overall PCa and significant disease as it had the highest net benefit at the majority of threshold probabilities along the *x*‐axis (Figure 3a,b). For the majority of threshold probabilities, the risk calculators performed better than a strategy of performing a biopsy for all patients or never performing a biopsy.

Using a risk threshold of 12.5% on the MRI‐ERSPC‐RC to identify which patients to biopsy, there would be a reduction of 106 transperineal biopsies. This 27% reduction in biopsies would have resulted in 29 missed ISUP grade group 1 cancers and 20 missed cases of significant cancers. Using a risk threshold of 20% to identify which patients to biopsy, the total number of biopsies performed would be reduced by 164. This 41.8% reduction in biopsies would have resulted in 51 missed ISUP grade group 1 cancers and 39 cases of significant cancers.

## 4 DISCUSSION

Deficiencies in contemporary PCa screening methods has led to a significant proportion of unnecessary prostate biopsies. Accordingly, there is an inherent need to develop methods to optimize patient selection prior to biopsy. Despite this, the selection of patients for biopsy remains controversial with no clear consensus among major urological guidelines.^18^, ^19^, ^20^, ^21^ The poor sensitivity of PSA testing has led to the European Association of Urology (EAU) guidelines on PCa recommending additional assessment with RCs or imaging to optimize patient selection.^18^ While other risk calculators and models exist, the MRI‐ERSPC‐RC, ERSPC‐RC, and PBCG‐RC have been recommended specifically by the EAU guidelines and are available online which enables easier access in clinical practice. Our study validated the MRI‐ERSPC‐RC in a contemporary population undergoing transperineal biopsy. We demonstrated that the new MRI‐ERSPC‐RC performed better than both the previous ERSPC‐RC and PBCG‐RC in predicting clinically significant PCa.

The use of the ERSPC‐RC with and without the addition of mpMRI as well as the PBCG‐RC was validated in our cohort for predicting the likelihood of clinically significant PCa. Overall, our population was younger, had a lower median PSA and prostate volume compared to the ERSPC cohort but similar in age and PSA to the PBCG cohort. The distribution of PI‐RADS scores were similar to the ERSPC cohort which had 82% of patients with PI‐RADS ≥ 3 mpMRI.^6^ Overall, we observed slightly poorer performance in our cohort compared to the original ERSPC population with Alberts et al reporting an AUC of 0.791‐0.839 for any PCa and 0.843‐0.850 for high‐grade PCa using the MRI‐ERSPC‐RC and an AUC of 0.708‐0.779 for any PCa and 0.742‐0.747 for high‐grade PCa with the ERSPC‐RC.^6^ Using the PBCG‐RC, we found a similar AUC for predicting high‐risk PCa with an AUC of 0.746 compared to 0.755 in the original study.^7^

Our results are comparable to other validation studies of the MRI‐ERSPC‐RC, ERSPC‐RC, and PBCG‐RC. Using the MRI‐ERSPC‐RC, Radtke et al, Pullen et al, Saba et al, and Mortezavi et al reported an AUC of 0.82‐0.84, 0.822, 0.85, and 0.87, respectively, for predicting clinically significant PCa.^22^, ^23^, ^24^, ^25^ The performance of the ERSPC‐RC was found to be poorer than the MRI‐ERSPC‐RC with Radtke et al, Mortezavi et al, Poyet et al, and Lundon et al describing an AUC of 0.77, 0.8, 0.73, and 0.69, respectively, for estimating the risk of significant PCa.^22^, ^25^, ^26^, ^27^ The performance of the PBCG‐RC has previously been evaluated with Saba et al identifying an AUC of 0.69, Mortezavi et al finding an AUC of 0.76, and Carbunaru et al identifying an AUC of 0.65 for the detection of clinically significant PCa.^24^, ^25^, ^28^ These studies were all performed in European or American populations, whereas our study was the first validation study performed in an Australian population. The consistency demonstrated across these validation studies with the present study suggests that these risk calculators may potentially be utilized in other populations of men for risk stratification of significant PCa.

The addition of mpMRI to the ERSPC‐RC improved the prediction of PCa of any grade and clinically significant PCa. mpMRI has been increasingly utilized with several studies demonstrating a higher rate of clinically significant PCa detection with pre‐biopsy mpMRI.^4^, ^29^, ^30^ Furthermore, a recent Cochrane Review suggested using an mpMRI‐driven biopsy pathway improved the diagnosis of Grade Group ≥ 2 PCa by 12% compared to systematic biopsy alone.^31^ These findings are consistent with our analyses suggesting that, while the ERSPC‐RC and PBCG‐RC were both effective in predicting high‐risk PCa, the inclusion of mpMRI data significantly improved the risk stratification.

The ability to quantify risk using RCs may enable the reduction of unnecessary biopsies. With the increased usage of mpMRI in the workup of PCa, a recent systematic review assessed the utility of mpMRI in predicting the absence of PCa. This review found that mpMRI alone could not reliably predict the absence of PCa, however, performance could be enhanced if used in conjunction with other methods that could quantify risk of PCa.^32^ Consequently, the use of RCs may enable improved patient selection for biopsy. Using a detectable cancer risk threshold to biopsy of ≥12.5% as recommended by the ERSPC group for the MRI‐ERSPC‐RC resulted in a 27% reduction in biopsies, 29 missed ISUP Grade Group 1 cancers, and 20 missed cases of significant cancers. It is recommended that follow‐up for patients with low MRI‐ERSPC‐RC risk scores should comprise serial PSA measurements and potential repeat mpMRI. Any change in PSA dynamics or suspicious lesions on an mpMRI would result in a higher score using the RC and patients should subsequently be considered for biopsy. While RCs should be used in standard practice to provide a quantifiable estimation of risk, it is important to recognize that RCs are designed as a tool to guide clinicians and patients in a shared decision‐making capacity and, ultimately, the decision to biopsy remains with the patient and his urologist.

Our study assessed the performance of the MRI‐ERSPC‐RC, ERSPC‐RC, and PBCG‐RC in a cohort undergoing transperineal prostate biopsy. However, the original RCs were developed from cohorts undergoing transrectal ultrasound‐guided (TRUS) biopsy.^6^, ^7^ There are inherent differences between transperineal and transrectal approaches and the use of targeted transperineal biopsies may have increased the cancer detection rate of significant cancer in our series. The validation studies of Radtke et al, Pullen et al, and Saba et al were all performed in a population undergoing transperineal prostate biopsy, whereas Mortezavi et al performed their study in a cohort undergoing TRUS biopsy.^22^, ^23^, ^24^, ^25^ Utilizing the MRI‐ERSPC‐RC, all of these studies found results consistent with our study with an AUC between 0.82 and 0.87 suggesting that, although the MRI‐ERSPC‐RC was developed in a TRUS biopsy cohort, its usage could be implemented in patients being considered for transperineal biopsy.

The ability to extrapolate the use of any predictive model to other populations is dependent on the prevalence of disease in the respective populations. Therefore, while our study validates the use of MRI‐ERSPC‐RC in an Australian population, our experience may not necessarily be shared with different populations with a large difference in PCa prevalence.

In conclusion, our study validates the ERSPC‐RC, MRI‐ERSPC‐RC, and PBCG‐RC in a cohort using transperineal prostate biopsy with the MRI‐ERSPC‐RC performing the best. Utilizing these RCs provides a risk prediction enabling improved shared decision making and can help to guide patient selection for biopsy.
